# Graft‐versus‐host disease, a rare complication after orthotopic liver transplantation

**DOI:** 10.1002/ccr3.1314

**Published:** 2017-12-07

**Authors:** Andrew Ofosu, Andrew Zabolotsky, Miguel Rufail, Joseph Spataro, Jesse Civan

**Affiliations:** ^1^ Division of Gastroenterology & Hepatology Department of Medicine Jefferson Digestive Health Institute Philadelphia Pennsylvania; ^2^ Department of Pathology, Anatomy, and Cell Biology Sidney Kimmel Medical College at Thomas Jefferson University Philadelphia Pennsylvania; ^3^ Department of Medicine Thomas Jefferson University Hospital Philadelphia Pennsylvania

**Keywords:** Esophageal erosions, graft‐versus‐host disease, hepatic sarcoidosis, liver cirrhosis, orthotopic liver transplantation

## Abstract

Acute graft‐versus‐host disease (GVHD) after orthotopic liver transplantation (OLT) is a rare but fatal complication that poses a major diagnostic and therapeutic challenge. Our case highlights the need for further studies to develop therapeutic modalities to improve outcomes in patients who develop GHVD following OLT.

## Case

A 70‐year‐old man underwent deceased donor orthotopic liver transplantation (OLT) for decompensated cirrhosis secondary to hepatic sarcoidosis. He presented to the emergency department on postoperative day 48 with a history of fever, an erythematous nonpruritic rash, and dysphagia to solids.

**Figure 1:**
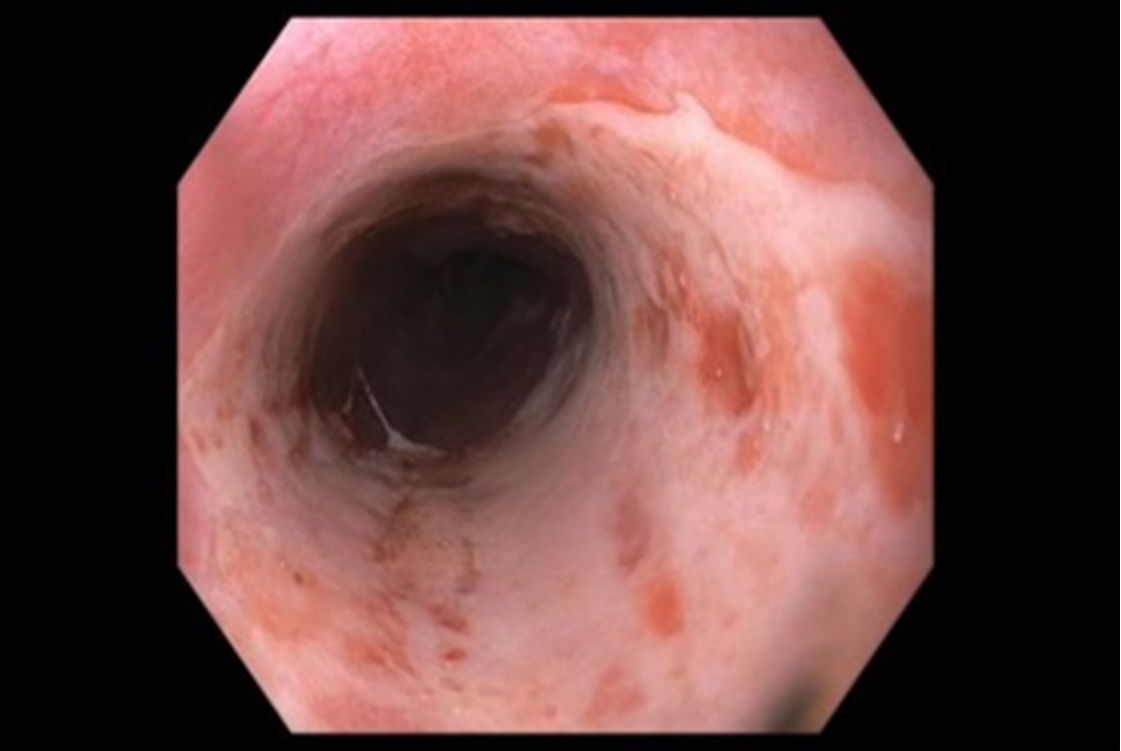
Diffuse, patchy, white erosions in the esophagus.

**Figure 2:**
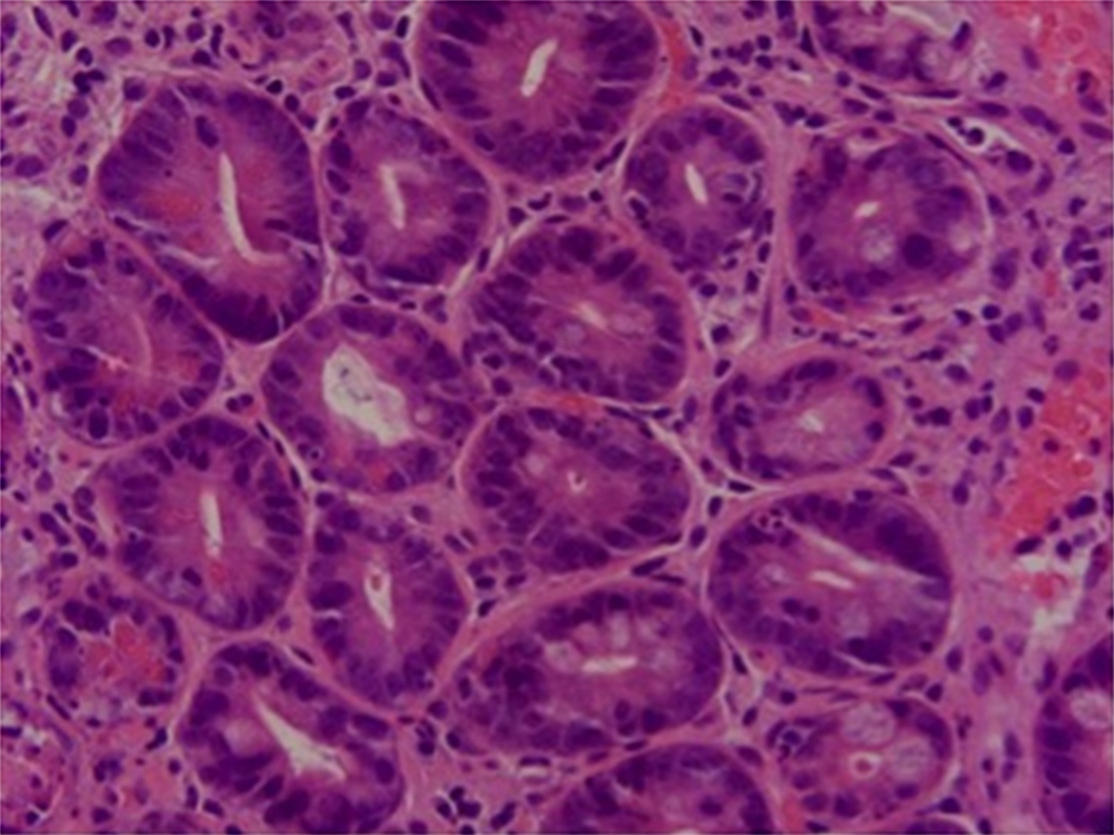
Extensive epithelial apoptosis with features compatible with grade III GVHD.

A skin biopsy showed acute vacuolar interface dermatitis with necrotic keratinocytes, consistent with graft‐versus‐host disease (GVHD). An upper endoscopy showed diffuse, patchy, white erosions in the esophagus (Fig. 1). Endoscopic duodenal biopsies showed mucosal erosions and extensive epithelial apoptosis with features compatible with grade III GVHD (Fig. 2).

Acute GVHD after OLT is a rare complication with an incidence of 0.1–2% and associated mortality rate greater than 75% 1, 2. A suggested underlying mechanism involves activation of immunocompetent donor lymphocytes originating from the transplanted liver graft inducing a destructive cellular immune response against recipient tissues 2.

Clinical symptoms confirmed with histopathological evidence or demonstration of chimerism should prompt early recognition and treatment. Due to the rarity of GVHD post‐OLT, most therapies are based on anecdotes and experiences treating GVHD following stem cell transplantation 2.

Despite treatment with methylprednisolone (3 mg/kg), antithymocyte globulin (40 mg/kg), and cyclosporine (10 mg/kg), our patient succumbed and died from sepsis. This highlights the need for further research to improve patient outcomes.

## Authorship

All authors: participated in drafting the article and revised it critically for important intellectual content, and approved the final version to be published.

## Conflict of Interest

None declared.
